# Outcomes of an Institutional Rapid Recovery Protocol for Total Joint Arthroplasty at a Safety Net Hospital

**DOI:** 10.5435/JAAOSGlobal-D-21-00173

**Published:** 2022-03-09

**Authors:** Adam J. Taylor, Robert D. Kay, Jason A. Bryman, Erik Y. Tye, Donald B. Longjohn, Soheil Najibi, Robert P. Runner

**Affiliations:** From the Department of Orthopaedic Surgery, Harbor-University of California, Los Angeles, Medical Center, Torrance, CA (Dr. Taylor, Dr. Kay, Dr. Bryman, and Dr. Tye); the Department of Orthopaedic Surgery, Rancho Los Amigos National Rehabilitation Center, Downey, CA (Dr. Taylor, Dr. Kay, Dr. Bryman, Dr. Tye, Dr. Longjohn, Dr. Najibi, and Dr. Runner); and the Department of Orthopaedic Surgery, Keck Medical School of University of Southern California, Los Angeles, CA (Dr. Longjohn).

## Abstract

Rapid recovery protocols (RRPs) for total joint arthroplasty (TJA) can reduce hospital length of stay (LOS) and improve patient care in select cohorts; however, there is limited literature regarding their utility in marginalized patient populations. This report aimed to evaluate the outcomes of an institutional RRP for TJA at a safety net hospital.

**Methods:**

A retrospective review of 573 primary TJA patients was done, comparing the standard recovery protocol (n = 294) and RRP cohorts (n = 279). Measured outcomes included LOS, 90-day complications, revision surgeries, readmissions, and emergency department visits.

**Results:**

The mean LOS reduced from 3.0 ± 3.1 days in the standard recovery protocol cohort to 1.6 ± 0.9 days in the RRP cohort (*P* < 0.001). The RRP cohort had significantly fewer 90-day complications (11.1% versus 21.4%, *P* = 0.005), readmissions (1.4% versus 5.8%, *P* = 0.007), and revision surgeries (1.4% versus 4.4%, *P* = 0.047).

**Conclusion:**

A RRP for primary TJA can be successfully implemented at a safety net hospital with a shorter LOS and fewer acute adverse events. Such protocols require a coordinated, multidisciplinary effort with strict adherence to evidence-based practices to provide high-quality, value-based surgical health care to an underserved cohort.

Primary total knee arthroplasty (TKA) and total hip arthroplasty (THA) are among the most effective, quality of life-improving procedures available to patients.^1^ Most patients reach the long-term goals of pain relief and restoration of function after total joint arthroplasty (TJA)^2^; however, these long-term outcomes may be overlooked by some patients because of acute postoperative pain and surgery-related morbidities.^3^ Because the demand for THA and TKA continues to increase,^4^ enchaining short-term outcomes has been a target of many surgeons through the use of rapid recovery protocols (RRPs), which aim to expedite recovery and reduce complications while maintaining the highest level of patient care.

Several authors have shown that RRP interventions, such as patient education, medical optimization, multimodal pain management,^5,6^ and early physical therapy, correspond to a shorter hospital length of stay (LOS) without increasing complication or readmission rates.^7,8,9,10^ Other studies have demonstrated that RRPs can be associated with reduced complication rates, improved functional outcomes, and improved range of motion after TKA.^11,12^ Moreover, because these protocols have been associated with lower costs and increased discharge home versus skilled nursing facilities,^13^ RRPs could be particularly valuable to safety net county hospitals where resources are limited and access to care is a particular challenge.

Critical to the success of a RRP, however, is patient selection, enlistment of strong social support, and the availability of perioperative hospital resources.^14,15^ Safety net hospitals are defined as those which “by mandate or mission deliver a large amount of care to uninsured and other vulnerable populations.”^16^ Unlike other cohorts, patients at safety net county hospitals tend to be of lower socioeconomic status with higher rates of housing insecurities, language disparities, comorbidities, and substance abuse.^17,18^ Patient cohorts in these safety net hospitals have also been shown to experience higher rates of arthritis,^19^ worse quality of life while awaiting surgery,^20^ longer hospital LOS, and more complications after TJA.^17,18,21^ Although these disparities create a unique set of challenges, the implementation of a standardized, multidisciplinary RRP could be of notable value in this cohort.

The purpose of this study was to evaluate the outcomes of primary TJA after the implementation of a RRP at a single safety net hospital because there is limited literature investigating these protocols at resource-limited institutions with marginalized patient populations. Schultz et al^8^ demonstrated that a RRP could be successfully implemented for a single surgeon at a county hospital; however, it has not been evaluated at an institutional level in the previous literature. The hypothesis was that a RRP for primary TJA could be safely and successfully implemented with no increase in complications and potentially benefit the system and patients with reduced hospital LOS.

## Methods

**Figure 1:**
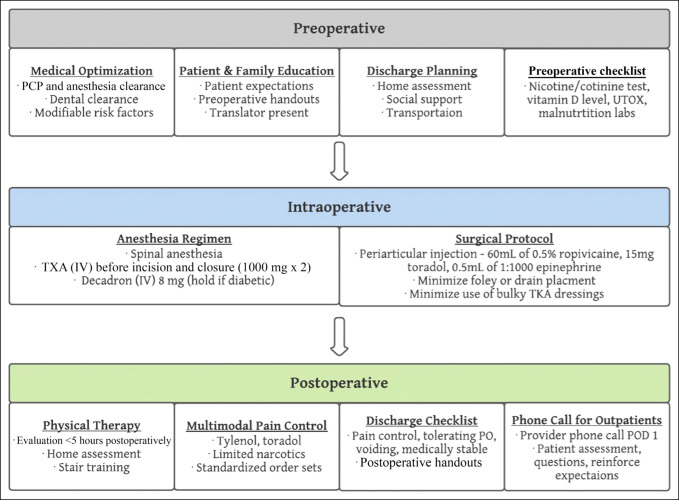
Flowchart illustrating the rapid recovery protocol. PCP = primary care physician, UTOX = urine toxicology screen, TXA = tranexamic acid, TKA = total knee arthroplasty, POD = postoperative day

On October 1, 2019, the senior author implemented a multidisciplinary RRP for TJA at an urban safety net hospital. The RRP was developed through coordination and input from orthopaedic surgeons, anesthesiologists, physical and occupational therapists, pharmacists, nurses, translators, and social workers. Highlights of this RRP included preoperative patient education handouts, medical and psychosocial optimization, increased usage of spinal anesthesia, opioid-sparing multimodal pain management techniques, intraoperative periarticular injections, intraoperative use of intravenous tranexamic acid (TXA), reduced usage of indwelling urinary catheters and closed suction drains, standardized order sets, and early mobilization with physical therapy on postoperative day (POD) 0 (Figure 1).

**Figure 2:**
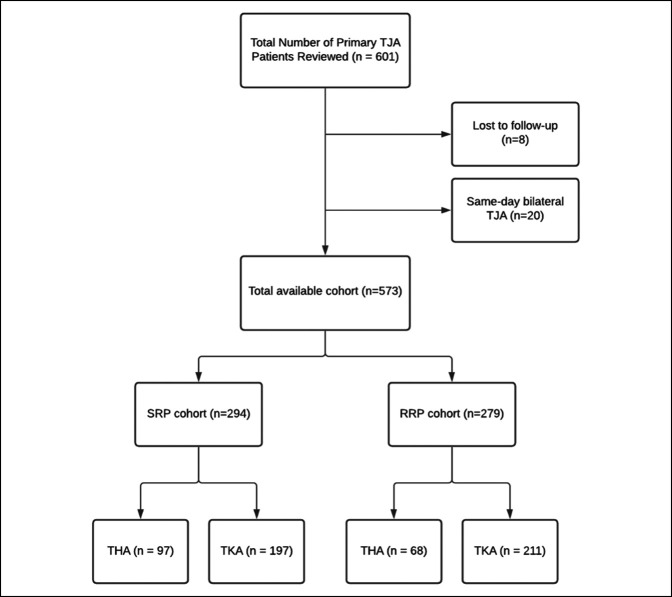
Flowchart depicting the inclusion criteria and cohort distribution. TJA = total joint arthroplasty, SRP = standard recovery protocol, RRP = rapid recovery protocol, THA = total hip arthroplasty, TKA = total knee arthroplasty

Institutional review board approval was obtained for the retrospective review of all primary TKA (Current Procedural Terminology [CPT] code 27477), THA (CPT code 27130), and conversion hip arthroplasty (CPT code 27132) patients treated from July 1, 2018, to October 31, 2020, at a single institution by four orthopaedic surgeons. Patients who had surgery before the initiation of the institutional RRP on October 1, 2019, were placed in the standard recovery protocol (SRP) cohort, and those treated after October 1, 2019, were placed in the RRP cohort. If a patient received staged bilateral procedures on separate hospitalizations, they were considered two separate procedures. Revision TJA, same-day bilateral TJA, or patients having less than 90 days of follow-up were excluded from this study. The resulting 279 patients in the RRP cohort were then compared against the previous 294 patients in the SRP cohort (Figure 2).

Primary outcome measures included hospital LOS and the number of midnights in the hospital. Secondary outcomes included discharge disposition, 90-day complications, 90-day revision surgeries, unplanned 90-day readmissions, and 30-day emergency department (ED) visits. All acute adverse events were diligently collected by chart review; these were organized and categorized according to a similar report by Schultz et al.^8^

All statistical analyses were completed with SPSS Statistics (version 10.15 for macOS; IBM) using a *P* value of 0.05. All continuous variables were analyzed using two sample Student *t*-tests, and all categorical data were analyzed by chi-square tests.

A total of 573 TJA patients over the 27-month period were included in this study. There were 294 patients (34.0% male and 66.0% female) in the SRP cohort and 279 (33.3% male and 66.7% female) in the RRP cohort. The most common ethnicity was Hispanic, comprising 75.9% in the SRP group and 76.3% in the RRP group (*P* = 0.211). Most of the patients were non-English speaking, with 78.9% in the SRP group and 76.7% in the RRP group (*P* = 0.547). Patient age, BMI, ASA classification, CCI, and preoperative diagnosis of DM or inflammatory arthritis were not found to be significantly different between groups (Table 1).

**Table 1:** Patient Demographics in the SRP Cohort and the RRP Cohort

	SRP Cohort (n = 294)	RRP Cohort (n = 279)
Age	61.26 ± 10.1	60.67 ± 10.33
Sex		
Male	34.0% (n = 100)	33.3% (n = 93)
Female	66.0% (n = 194)	66.7% (n = 186)
BMI	30.63 ± 4.78	30.99 ± 4.52
Self-reported race		
Hispanic	75.9% (n = 223)	76.3% (n = 213)
African American	9.2% (n = 27)	12.9% (n = 36)
White	7.1% (n = 21)	6.8% (n = 19)
Asian	6.5% (n = 19)	2.9% (n = 8)
Others	1.4% (n = 4)	1.0% (n = 3)
Primary language		
English	21.1% (n = 62)	23.3% (n = 65)
Non-English	78.9% (n = 232)	76.7% (n = 214)
ASA classification	2.33 ± 0.59	2.39 ± 0.56
I	6.1% (n = 18)	3.5% (n = 2)
II	54.4% (n = 160)	53.8% (n = 83)
III	39.5% (n = 116)	42.7% (n = 39)
CCI	2.39 ± 1.42	2.45 ± 1.57
Diabetes mellitus	25.2% (n = 74)	28.3% (n = 79)
Preoperative HbA1c	6.60 ± 0.96	6.54 ± 0.57
Inflammatory arthritis	11.2% (n = 33)	10.0% (n = 28)
Smoking status		
Never smokers	73.1% (n = 215)	76.7% (n = 214)
Former smokers	23.5% (n = 69)	23.3% (n = 65)
Current smokers	3.4% (n = 10)	0.0% (n = 0)

ASA = American Society of Anesthesiology, BMI = body mass index, CCI = Charlson Comorbidity Index, HbA1c = hemoglobin A1c, RRP = rapid recovery protocol, SRP = standard recovery protocol, VAS = visual analog scale

The 294 patients in the SRP cohort included 97 THAs (33.0%) and 197 TKAs (67.0%), and the 279 patients in the SRP cohort included 68 THAs (24.3%) and 211 TKAs (75.6%) (Table 2). Patients in the RRP group were more likely to have spinal anesthesia (64.2% versus 3.1%) and be the first case of the day (62.0% versus 51.7%). Patients in the RRP group were less likely to have closed suction drain placement (20.4% versus 94.5%) and indwelling urinary catheterization (39.0% versus 98.0%).

**Table 2:** Surgical Characteristics in the SRP Cohort and the RRP Cohort

	SRP Cohort (n = 294)	RRP Cohort (n = 279)
THA (CPT code 27130, 27132)	33.0% (n = 97)	24.3% (n = 68)
Conversion (CPT code 27132)	9.3% (n = 9)	13.2% (n = 9)
Cemented	7.3% (n = 7)	2.9% (n = 2)
TKA (CPT code 27477)	67.0% (n = 197)	75.6% (n = 211)
Cemented	29.4% (n = 85)	32.3% (n = 90)
CCK	7.6% (n = 15)	5.7% (n = 12)
Staged bilateral procedures	22.1% (n = 65)	20.8% (n = 58)
Type of anesthesia		
Spinal	3.1% (n = 9)	64.2% (n = 179)
General endotracheal	96.9% (n = 285)	35.8% (n = 100)
Closed suction drain	94.5% (n = 279)	20.4% (n = 57)
Indwelling urinary catheter	98.0% (n = 288)	39.0% (n = 109)
First case of the day	51.7% (n = 152)	62.0% (n = 173)

CCK = constrained condylar knee, EBL = estimated blood loss, RRP = rapid recovery protocol, SRP = standard recovery protocol, THA = total hip arthroplasty, TKA = total knee arthroplasty

## Results

### Length of Stay and Discharge Disposition

The mean LOS was significantly reduced from 3.0 ± 3.1 days in the SRP cohort to 1.56 ± 0.9 days in the RRP cohort (*P* < 0.001) (Table 3). No patients were observed in the SRP group who were discharged on the day of surgery compared with 60 patients (21.5%) in the RRP group (*P* < 0.001).

**Table 3:** LOS and Discharge Disposition in the Pre-COVID versus Post-COVID Groups

	SRP Cohort (n = 294)	RRP Cohort (n = 279)	*P*
Hospital LOS (d)	2.97 ± 3.11	1.59 ± 0.90	<0.001^a^
Same-day discharge	0.0% (n = 0)	21.5% (n = 60)	<0.001^a^
No. of midnights in the hospital			<0.001^a^
0	0.0% (n = 0)	21.5% (n = 60)	
1	3.7% (n = 11)	42.3% (n = 118)	
2	69.0% (n = 203)	29.7% (n = 83)	
3	19.4% (n = 57)	4.7% (n = 13)	
4	3.1% (n = 9)	1.1% (n = 3)	
≥5	4.8% (n = 14)	0.7% (n = 2)	
Discharge disposition			0.681
Home	97.3% (n = 286)	98.9% (n = 276)	
SNF	0.3% (n = 1)	0.4% (n = 1)	
AIR	1.4% (n = 4)	0.7% (n = 2)	
Recuperative care	1.0% (n = 3)	0.0% (n = 0)	

AIR = acute inpatient rehabilitation, LOS = length of stay, RRP = rapid recovery protocol, SNF = skilled nursing facility, SRP = standard recovery protocol

a *P* < 0.05.

### Surgical Outcomes and Acute Adverse Events

A significant reduction was observed in the total surgery time (164.5 versus 175.6 minutes, *P* < 0.001), mean EBL (171.0 versus 268.0 mL, *P* < 0.001), and requirement for blood transfusion postoperatively (1.8% versus 5.8%, *P* = 0.016) in the RRP group.

Patients from the RRP cohort had significantly fewer complications (11.1% vs 21.4%, *P* = 0.005), 90-day readmissions (1.4% vs 5.8%, *P* = 0.007), and 90-day revision surgeries (1.4% vs 4.4%, *P* = 0.047) (Table 4). For postoperative complications specifically, there was a significant decrease in acute surgical complications (1.1% versus 4.1%, *P* = 0.003) and acute medical complications (3.6% versus 9.9%, *P* = 0.003) in the RRP cohort. A significant reduction was observed in the total surgery time (164.5 versus 175.6 minutes, *P* < 0.001), mean EBL (171.0 versus 268.0 mL, *P* < 0.001), and requirement for blood transfusion postoperatively (1.8% versus 5.8%, *P* = 0.016) in the RRP group.

**Table 4:** Surgical Outcomes and Acute Adverse Events

	SRP Cohort (n = 294)	RRP Cohort (n = 279)	*P*
Total surgery time (min)	175.6 ± 36.4	164.5 ± 32.7	<0.001^a^
EBL (mL)	268.0 ± 200.1	171.0 ± 175.0	<0.001^a^
Postoperative blood transfusion	5.8% (n = 17)	1.8% (n = 5)	0.016^a^
Any 90-day complication	21.4% (n = 63)	11.1% (n = 31)	0.005^a^
Acute surgical complications	4.1% (n = 12)	1.1% (n = 3)	0.003^a^
Acute medical complications	9.9% (n = 29)	3.6% (n = 10)	0.003^a^
Superficial wound complications	6.1% (n = 18)	6.1% (n = 17)	1.000
Deep wound complications	1.4% (n = 4)	0.4% (n = 1)	0.374
30-day ED visits	4.4% (n = 13)	2.5% (n = 7)	0.258
90-day readmissions	5.8% (n = 17)	1.4% (n = 4)	0.007^a^
90-day revision surgeries	4.4% (n = 13)	1.4% (n = 4)	0.047^a^

ED = emergency department, RRP = rapid recovery protocol, SRP = standard recovery protocol

a *P* < 0.05.

In the SRP group, there were a total of 63 complications (21.4%), 17 unplanned readmissions (5.8%), 13 ED visits (4.4%), and 13 revision surgeries (4.4%). There were 12 acute surgical complications (4.1%), including intraoperative calcar fractures (2.0%, n = 6), aseptic loosening (0.7%, n = 2), intraoperative acetabular fracture (0.3%, n = 1), acute postoperative tibial tubercle fracture (0.3%, n = 1), retained closed suction drain (0.3%, n = 1), and a popliteus artery injury, which was acutely repaired by the vascular surgery team (0.3%, n = 1). There were 29 acute medical complications (9.9%) found, including postoperative anemia requiring transfusion (5.8%, n = 17), pulmonary embolism (1.0%, n = 3), urinary retention requiring catherization (1.0%, n = 3), deep vein thrombosis (0.3%, n = 1), urinary tract infection (0.3%, n = 1), CHF exacerbation (0.3%, n = 1), postoperative supraventricular tachycardia (0.3%, n = 1), hypertensive urgency (0.3%, n = 1), and a first-degree atrioventricular block (0.3%, n = 1). There were 18 superficial wound complications found (6.1%), 13 of which were treated with local wound care (72.2%) and 5 of which returned to the operating room for superficial débridement and scar revision (27.8%) (POD 35, 42, 62, 70, and 84). There were four acute periprosthetic joint infections (n = 1.4%), all of which underwent débridement, antibiotics, and implant retention (DAIR) procedure (POD 14, 16, 28, and 56), one of which had a recurrent infection and underwent a two-stage antibiotic spacer placement on POD 76.

In the RRP group, there were 31 complications (11.1%), four unplanned readmissions (1.4%), seven ED visits (2.5%), and four revision surgeries (1.4%). The complications included three acute surgical complications (1.1%), including THA dislocation (0.4%, n = 1), intraoperative patellar tendon avulsion (0.4%, n = 1), and intraoperative calcar fracture (0.4%, n = 1). There were 10 acute medical complications (3.6%), including postoperative anemia requiring transfusion (1.8%, n = 5), sepsis secondary to a retroperitoneal abscess found on POD 22 (0.4%, n = 1), pulmonary embolism (0.4%, n = 1), postoperative hypotension (0.4%, n = 1), acute kidney injury (0.4%, n = 1), and one patient deceased on POD 5 from complications related to an acute small bowel obstruction (0.4%, n = 1). There were 17 superficial wound complications (6.1%), 14 of which healed with local wound care (82.4%) and three of which returned to the operating room for superficial débridement and scar revision (17.6%) (POD 29, 41, and 47). There was one deep wound complication (0.4%) secondary to an acute hematogenous periprosthetic joint infection which underwent a DAIR procedure on POD 55 without additional issues to date.

## Discussion

Enhancing the short-term outcomes of TJA through the implementation of RRPs has gained notable interest over the past 15 years. Several studies have now demonstrated the safety, efficacy, and cost-saving potential of RRPs for TJA in select patient cohorts and hospital systems.^7,8,9,10,11,12,22-24^ Limited literature exists, however, regarding the utility of implementing a RRP in more marginalized patient populations and at resource-limited facilities. This study demonstrates that an institutional RRP can be successfully implemented at a safety net hospital with reduced LOS while still achieving fewer complications, readmissions, ED visits, and revision surgeries.

Similar to other authors, the RRP in the present series consisted of a multidisciplinary approach that involves not only orthopaedic surgeons but also referring providers, anesthesiologists, nursing, physical and occupational therapists, social workers, pharmacists, and hospital administrators.^6,8,24^ Through these coordinated efforts, hospital LOS was reduced from 3.0 to 1.6 days (*P* < 0.001), which is now well below the national average of 2.8 days for TJA.^25^ In addition, 60 patients (21.5%) in the RRP were discharged home on the day of surgery, all of which occurred during the final 6 months of this series. Furthermore, the proportion of same-day discharges in this series is approximately three times that of the only other similar study reporting on a RRP for TJA patients in a county population (21.5% versus 6.5%).^8^ Perhaps more importantly, the reduction in LOS in this series was achieved while still maintaining lower rates of 90-day complications, readmissions, and revision surgeries. Because reimbursements for TJA transition to bundled payments and Medicare continue to incentivize early discharge by decreasing reimbursement for each hospital day,^25^ lowering LOS and complication rates becomes essential to determining successful changes in RRPs, even in marginalized patient populations.

Although ASA and CCI were not found to be markedly different between groups in this series, the reduction in complications, particularly acute medical complications, may represent improvements in medial and psychosocial optimization. The higher rates of substance abuse and comorbidities present in safety net hospital systems have been previously shown to negatively affect LOS and complications after TJA.^17,21^ Therefore, as part of the RRP, patients were required to be not currently smoking and complete a preoperative sobriety pathway if they had a history of alcohol or illicit drug abuse. In addition, because preoperative opioid use has been associated with a longer LOS and increased cost of care and negatively affect patient-reported outcomes after TJA,^26-28^ patients in the RRP group were required to be off all preoperative opioids before scheduling surgery. Postoperatively, a multimodal, opioid-sparing pain management regimen was used, which has demonstrated success in improving postoperative clinical outcomes and patient satisfaction, promoting a faster return of function, lowering hospital LOS, and reducing opioid-related adverse effects after TJA.^6,29,30,31,32^

An additional factor critical to the success of the RRP in this series was intensive patient education and communication. Unlike other cohorts, however, most patients at the authors' institutions are non-English speaking, comprising 76.7% of the RRP cohort. For this reason, in-person translators were made available during all patient encounters to enhance communication and patient understanding. In addition, preoperative and postoperative patient education handouts were constructed and provided to patients in their primary language. Patients who were discharged on the day of surgery were called by a provider on POD 1 to assess the pain level and functional status and answer any additional questions that may have arisen postsurgically, which may have reduced the number of avoidable ED visits.

Perioperatively, several changes were implemented as part of the surgical protocol, which may have further contributed to the improvements in LOS and postoperative complication rates. As one of the main portions of the RRP pathway, spinal anesthesia was used more frequently than general endotracheal anesthesia in the RRP group (64.2% versus 3.1%). Previous studies have demonstrated an increase in unforeseen overnight admissions due to morbidities associated with general endotracheal anesthesia, such as orthostatic hypotension, urinary retention, and nausea.^33,34^ Although 35.8% of the patients in the RRP group underwent general endotracheal anesthesia, this proportion continued to decrease because the protocol became more established and providers became more facile with performing spinal anesthesia. Furthermore, there was a notable reduction in the usage of indwelling catheters in the RRP group (39.0% versus 98.0%), which has been correlated with increased postoperative urinary traction infections, higher hospital costs, and decreased postoperative ambulation distance after TJA.^35,36^ In addition, the use of closed suction drains markedly decreased in the RRP group (20.4% versus 94.5%), which has been previously correlated with a greater need for transfusion postoperatively.^37,38^ To further limit blood loss and transfusion requirement, intravenous TXA was also administered TXA intraoperatively.^39^ Ultimately, the success of the RRP in this series is multifactorial and a representation of several institutional changes based on evidence-based practices.

There are limitations to this study, including the standard limitations of retrospective cohort analysis. This series was limited to a total of 573 TJA procedures; however, this is roughly three times the number of patients included in the only other study on RRP for TJA in a county population.^8^ In addition, this study is strengthened by a low attrition rate for this analysis, with only eight patients (1.4%) lost to follow-up in this series. Although this series reported only on 90-day postoperative outcomes, this data set does have a reasonable short-term follow-up for the relevant early complications associated with TJA procedures. In addition, insufficient data were collected to compare any patient-reported or functional outcomes, which, although was not the primary goal of this study, would be an important area of focus for future research. Patients in this series may also have presented to outside hospital EDs during the follow-up period, which may have led to inaccuracies in the exact number of ED visits reported; however, this is limited in county patient populations who often seek care at the local county facility, given their payor limitations. Finally, direct assessment of the exact difference in hospital or procedural costs between cohorts was not available, although it may be presumed to decrease in a similar manner that has been reported in comparable studies, given the reduction in LOS and complications.^8,10,13^ Nonetheless, this is still the largest and most comprehensibly measured cohort, to our knowledge, assessing the outcomes of an institutional RRP for TJA at a safety net hospital. Future research is necessary to compare the long-term complication rates among these cohorts.

## Conclusion

In a safety net hospital, a RRP for TJA can be safely and successfully implemented. Although patient-related and hospital-related disparities provided a unique set of challenges, this protocol demonstrated a shorter LOS while still maintaining lower 90-day complication, readmission, and revision surgery rates. Similar to other RRPs, process standardization and adherence to evidence-based practices through coordinated, multidisciplinary efforts were necessary. Because we transition into an era focused on value-based arthroplasty, an additional development of these protocols will become an important element to provide high-quality surgical care to an underserved cohort.
